# Impact of physical exercise habit on career decision-making behavior in college students: a chain mediating effect of self-efficacy and psychological resilience

**DOI:** 10.3389/fpsyg.2025.1647860

**Published:** 2025-08-19

**Authors:** Chuanjie Niu, Deng Jiaxin, Yongfeng Liu

**Affiliations:** School of Sports Training, Chengdu Sport University, Chengdu, China

**Keywords:** physical exercise, self-efficacy, psychological resilience, career decision-making, chain mediation effect

## Abstract

**Objective:**

To explore the mechanism through which physical exercise habits influence college students' career decision-making behavior, and to verify the independent and chain mediation effects of self-efficacy and psychological resilience within this relationship.

**Methods:**

Using stratified random sampling, questionnaires were distributed to universities students across China, and valid questionnaires were collected. The Physical Activity Rating Scale (PARS-3), General Self-Efficacy Scale (GSES), Connor-Davidson Resilience Scale (CD-RISC-10), and Career Decision-Making Difficulty Scale (CDMP) were employed. Structural equation modeling and the Bootstrap method were used to test the chain mediation effect.

**Results:**

Physical exercise habits significantly and positively influenced career decision-making ability. Both self-efficacy and psychological resilience served as independent mediators. The chain mediation path from self-efficacy to psychological resilience was significant. The mediation effects accounted for 39.6% of the total effect.

**Conclusion:**

Physical exercise significantly and positively influences career decision-making behavior by enhancing self-efficacy and psychological resilience, with self-efficacy and psychological resilience forming a chain transmission mechanism.

## 1 Introduction

With the development of the global economy, energy transition, and rapid technological changes, competition in the job market is intensifying (Bagga, 2023; Hanson, 2023; Autor et al., 2023). As a result, career decision-making has become a core challenge for college students. Consequently, career decision-making has become a core challenge for college students. Career decision-making concerns the specific path of an individual's future career development. Scholars such as Jeong, Lee, and Parola have also found it to be closely related to mental health, social adaptability, and life satisfaction (Jeong, 2020; Lee and Jung, 2022; Parola and Marcionetti, 2022). In recent years, physical exercise behavior, as an important vehicle for cultivating non-cognitive abilities, has gradually garnered academic attention for its impact on students' mental health. Research by Ouyang and Glavaš indicates that regular physical exercise has a significant positive effect on enhancing individuals' self-efficacy (Ouyang et al., 2020; Glavaš et al., 2024); Researchers such as Qiu and Li have also confirmed a positive correlation between maintaining physical exercise habits and enhancing psychological resilience in adolescents (Qiu et al., 2025; Li N. et al., 2024). These traits enhance individuals' ability to handle uncertainty and make informed career choices. Self-efficacy and psychological resilience are precisely key psychological resources in the individual career decision-making process (Santos et al., 2018; Pang et al., 2021). However, existing research mostly focuses on the main effects of physical exercise on academic performance or mental health; systematic exploration of how it transmits to the field of career decision-making through psychological mechanisms remains insufficient. Regarding the impact of physical exercise habits on college students' career decision-making behavior, current research mainly has the following two limitations: First, career decision-making research often emphasizes cognitive factors (e.g., interests, information processing) while overlooking non-cognitive factors (Hirschi et al., 2015). Second, most studies exploring psychological benefits of physical exercise focus on single mediators and neglect potential chain or synergistic effects between traits.

This study examines (1) whether physical exercise habits influence career decision-making behavior, (2) the independent mediating roles of self-efficacy and psychological resilience, and (3) the potential chain mediation between these traits. This study hopes to provide a scientific basis for multi-dimensionally optimizing college students‘ career decision-making behavior by analyzing the “physiological-psychological-behavioral” transmission mechanism. Theoretically, this study integrates self-efficacy and resilience theories to construct a model explaining how physical exercise habits influence career decision-making. Practically, the findings may help universities optimize career guidance programs. Based on literature review and theoretical deduction, the study proposes three hypotheses: H1, physical exercise habits significantly and positively influence college students' career decision-making ability; H2, self-efficacy and psychological resilience independently mediate the relationship between physical exercise and career decision-making; H3, there exists a chain mediation effect involving self-efficacy and psychological resilience.

## 2 Theoretical framework construction

### 2.1 Relationship between physical exercise habits, self-efficacy, and psychological resilience

Albert Bandura, the founder of self-efficacy theory, defined self-efficacy as an individual's subjective assessment of their ability to complete tasks, and this assessment can influence behavioral motivation (Bandura and Wessels, 1997). Early researchers generally defined psychological resilience as an individual's ability to effectively adapt, recover, and maintain psychological and emotional health when facing stress, adversity, trauma, or significant challenges (Masten, 2001; Luthar et al., 2000; Bonanno et al., 2004). Existing research indicates that physical exercise habits significantly promote the development of individuals‘ non-cognitive abilities, including self-efficacy and psychological resilience. Researchers such as Cao found that regular physical exercise can enhance individuals' self-efficacy by increasing their sense of bodily control and experiences of goal achievement (Cao et al., 2025). Specifically, the process of overcoming challenges in sports helps individuals form positive evaluations of their own abilities, for instance, improving personal accomplishment and positive performance feedback, thereby strengthening their belief in their capacity to achieve goals (Glavaš et al., 2024). Regarding psychological resilience, researchers like Qiu found in a meta-analysis that physical exercise can enhance psychological resilience through physiological mechanisms such as regulating stress hormone secretion and improving neuroplasticity, stress tolerance, emotional regulation (Qiu et al., 2025). Concurrently, studies have found that individuals who maintain long-term exercise habits have a higher proportion of tendencies to view setbacks as opportunities for growth. By enhancing psychological pathways such as stress tolerance and emotional regulation, they exhibit stronger adaptability in stressful environments (Li S. et al., 2024). Therefore, physical exercise habits may enhance self-efficacy and psychological resilience through parallel pathways of physiological activation and behavioral reinforcement.

### 2.2 Relationship between self-efficacy and psychological resilience

Based on Bandura's social cognitive theory (Locke, 1987), there exists a dynamic chain reaction mechanism between self-efficacy and psychological resilience. (1) The impact of self-efficacy on cognitive assessment and stress management exists. As an individual's belief in their own abilities, self-efficacy significantly affects their cognitive assessment and behavioral choices in stressful situations. Individuals with high self-efficacy tend to view challenges as manageable opportunities when faced with career decision-making pressure, and therefore adopt proactive problem-solving strategies rather than passive avoidance (Lee and Jung, 2022). (2) There is the role of adaptive attribution and perceived control. Santos et al. found that self-efficacy provides a key cognitive foundation for psychological resilience by influencing individuals' attribution styles (such as attributing difficulties to modifiable external factors rather than stable personal defects) and enhancing their sense of control over stressful situations (Santos et al., 2018). This adaptive attribution and control belief helps maintain psychological resilience in the face of setbacks and may regulate the emotional intensity of stress responses. (3) The cumulative effect of resilience should not be ignored. The proactive coping model not only effectively alleviates the negative impact of stress, but also further strengthens individuals' adaptability through successful coping experiences, forming a positive cycle of enhancing psychological resilience (Lee and Jung, 2022; Santos et al., 2018). Therefore, self-efficacy and psychological resilience together form a psychological resource pool that supports career decision-making behavior, and there may be a chain mediated effect between the two. These psychological processes play a central role when individuals face uncertainties related to career decision-making.

### 2.3 Relationship between self-efficacy, psychological resilience, and career decision-making

Career decision-making behavior, as a complex psychological-behavioral process, is directly influenced by an individual's psychological resource system, with self-efficacy and psychological resilience playing key roles. From the perspective of Social Cognitive Career Theory (SCCT), the influence of self-efficacy on career decision-making is mainly reflected in three aspects (Jeong, 2020; Hirschi et al., 2015). (1) Efficient information processing. Self-efficacy optimizes decision-making quality by enhancing information processing efficiency. Individuals with high self-efficacy exhibit more systematic career information search patterns and can integrate and analyze various types of career information more effectively (Hirschi et al., 2015). This enhanced cognitive ability gives them greater insight when evaluating career options, enabling them to weigh the pros and cons of different choices more accurately. (2) Reduction of decision-making anxiety. Self-efficacy significantly reduces anxiety levels during the decision-making process. Research shows that college students with higher career decision-making self-efficacy exhibit lower decision-making anxiety; this emotional advantage allows them to maintain a more rational decision-making state (Jeong, 2020). This emotional regulation function is particularly important when facing significant career choices. (3) Clear and achievable goal setting. Self-efficacy enhances the clarity and achievability of career goal setting. Individuals with high self-efficacy can not only set more challenging career goals but also break these goals down into specific action plans, thereby enhancing the execution of decisions (Jeong, 2020). The influence of psychological resilience on career decision-making is mainly manifested in two levels: stress coping and action maintenance. (1) Coping with uncertainty. Psychological resilience helps individuals cope with the uncertainty inherent in the career decision-making process by constructing an effective stress buffering mechanism. This buffering effect manifests as reducing the perceived threat of uncertainty, increasing tolerance for career risks, and maintaining psychological stability during the decision-making process (Parola and Marcionetti, 2022). These mechanisms work together to significantly reduce decision-making procrastination caused by fear of difficulties. (2) Maintaining behavioral engagement. Psychological resilience ensures the continuity of decision-making behavior. When facing decision-making obstacles or initial setbacks, individuals with strong psychological resilience can maintain behavioral engagement and avoid premature abandonment. This trait of persistence compensates for the limitations of relying solely on self-efficacy (Fletcher, 2013). It is noteworthy that, as mentioned earlier, the two do not operate independently. When an individual possesses both high self-efficacy and high psychological resilience, their career decision-making behavior may exhibit a dual-path reinforcement characteristic: self-efficacy drives the individual's decision-making actions, while psychological resilience ensures the persistence of those actions (Fletcher, 2013; Yong-Ti and Ling, 2018).

### 2.4 Theoretical framework model

**Figure 1:**
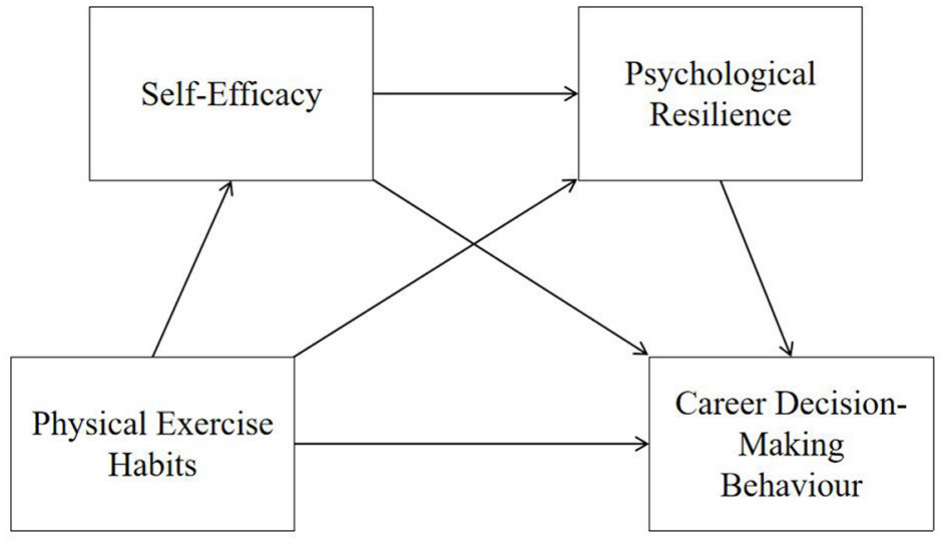
Chain mediation theoretical framework model.

Based on the above analysis, this study constructs the framework model shown in Figure 1. Figure 1 illustrates a chain mediation model where physical exercise habits influence self-efficacy, which in turn improves psychological resilience, ultimately enhancing career decision-making behavior.

## 3 Objects and methods

This study used mature scales published by previous researchers, and the content did not involve sensitive information or privacy infringement. According to the guidelines of ethical approval agencies and Chinese laws and regulations, this study was reviewed and approved by the Ethics Committee of the School of Sports Training of Chengdu Sport University (approval number: CTYLL2025007). All participants voluntarily participated and signed a written informed consent form, clearly understanding the research purpose, process, potential risks, and rights. The personal information and data collected during the research process are processed anonymously, used only for academic analysis, and strictly adhere to confidentiality principles to ensure the privacy and security of participants, without causing any negative impact on their physical and mental health. This study emphasizes that explaining the topic of filling out the questionnaire, the clear meaning of each question, and the content of the informed consent form to participants is a crucial step that cannot be ignored. We explained the clear meaning of each question in the questionnaire to each participant.

### 3.1 Research objects

This study focuses on the impact of physical exercise habits on college students' career decision-making behavior under the chain mediation effect of self-efficacy and psychological resilience. The survey subjects were undergraduate students enrolled in universities nationwide in China. According to the 2021 National Education Development Statistical Bulletin released by the Ministry of Education of the People's Republic of China, there were currently 19.0654 million undergraduate students in China (*N* > 100,000) (Ministry of Education of the People's Republic of China, 2022). Following the survey guidelines issued by the U.S. National Center for Health Statistics (NCHS), a population larger than 100,000 qualifies as a very large population in statistics (Akinbami et al., 2022). Therefore, Cochran's sample size calculation formula for proportions under very large populations was applied: $n=\frac{Z^{2}\times p\times \left(1-\;p\right)}{e^{2}}$. Parameters were set at a 95% confidence interval (*Z* = 1.96), a 5% margin of error (e = 0.05), and the most conservatively estimated expected proportion *p* = 0.5, the formula after inserting the data is $n=\frac{1.96^{2}\times 0.5\times \left(1-\;0.5\right)}{0.05^{2}}$. Substituting the data yielded a sample size of 384, meaning at least 384 valid questionnaires needed to be collected (Cochran, 1977). According to Nulty's research, the average valid response rate for online surveys among student groups ranges from 40% to 60%. To ensure the validity of this study, the number of questionnaires distributed should be no < 960 (Nulty, 2008). Using stratified random sampling based on student age and major, 1000 questionnaires were distributed to undergraduate students from 15 provinces and municipalities across the country. This research divided the target population according to specific geographical locations, covering as many survey subjects as possible from various geographical locations in China (Northeast, Northwest, North and Central China, Southwest, Southeast, etc.). However, factors such as gender, age, and major were not deliberately stratified. Six hundred and ninty-six questionnaires were returned, after removing the survey questionnaires with missing data and other issues, 412 valid questionnaires were obtained, resulting in a valid return rate of 41.20%. The basic information of the survey subjects is shown in Table 1.

**Table 1:** Basic information of survey subjects.

**Basic information**	**Category**	**Population**	**Proportion**
Gender	Male	228	55.34%
	Female	184	44.66%
Age	18–19	69	16.75%
	19–20	68	16.50%
	20–21	85	20.63%
	21–22	91	22.09%
	22–23	72	17.48%
	23–24	27	6.55%
Major	Science & Eng.	96	23.30%
	Economics	54	13.11%
	Literature	53	12.86%
	Art	32	7.77%
	Education	67	16.26%
	Medicine	31	7.52%
	Law	37	8.98%
	Political research	20	4.85%
	Sports	22	5.34%

### 3.2 Measurement tools

#### 3.2.1 Physical Activity Rating Scale (PARS-3)

The Physical Activity Rating Scale (PARS-3) was selected from the research of Chinese scholar Deqing (1994). This scale was revised based on physical activity questionnaires proposed by scholars such as Baecke et al. (1982) and Godin and Shephard (1985) and adjusted to suit the characteristics of physical activity among Chinese students. The scale consists of three items assessing indicators across three dimensions: exercise intensity, duration, and frequency. Items one to three correspond to the intensity, duration, and frequency of the participant's physical activity, respectively. Items one and three are scored from 1 to 5 (options 1–5). While item two is scored from 0 to 4 (options 1–5), this is because project 2 evaluates individual exercise duration, and excessively low exercise duration (option 1) is considered almost ineffective, therefore 0 points are awarded. The total score is calculated by multiplying the scores of items one to three: Intensity × Time × Frequency, to comprehensively assess the physical activity level of participants. Assuming participants choose option 3 (3 points) in the first question, option 2 (1 point) in the second question, and option 1 (1 point) in the third question, the total score is 3 × 1 × 1 = 3 points. In this study, the scale's reliability was 0.803 and its validity was 0.713, indicating that the scale reliably and effectively measured the target variable in this study, supporting the scientificity and credibility of the findings (see Table 2).

**Table 2:** Reliability and validity tests of scales.

**Index**	**PARS-3**	**GSES**	**CD-RISC-10**	**CDMP**
Cronbach α	0.80	0.97	0.94	0.96
KMO	0.71	0.98	0.96	0.98

KMO values above 0.70 indicate sampling adequacy for factor analysis.

#### 3.2.2 General Self-Efficacy Scale (GSES)

The General Self-Efficacy Scale (GSES) was developed by German psychologists Schwarzer and Jerusalem (1995). The scale was designed based on Bandura's self-efficacy theory (Bandura and Wessels, 1997). In this study, the scale was translated into Chinese to provide a clearer understanding of its contents for the research subjects. The scale consists of 10 items, is unidimensional (no subscales), and aims to measure the overall level of participants' self-efficacy. For each item, the four options “Completely Disagree,” “Occasionall*y* Agree,” “Fairly Agree,” and “Strongly Agree” are scored 1–4 points, respectively. The total score for all items is summed to obtain the participant's overall self-efficacy score. In this study, the scale's reliability was 0.968 and its validity was 0.976, indicating that the scale reliably and effectively measured the target variable in this study, supporting the scientificity and credibility of the findings (see Table 2). The high reliability and validity of this scale may be due to sample homogeneity and the single-dimensional structure of the scale.

#### 3.2.3 Connor-Davidson Resilience Scale (CD-RISC-10)

The Connor-Davidson Resilience Scale (CD-RISC-10) was proposed by scholar Campbell-Sills and Stein (2007), refined and improved based on the 25-item resilience scale developed by Connor and other researchers (Connor and Davidson, 2003). This study used the CD-RISC-10 scale instead of the original CD-RISC-25 scale because it has undergone refinement and improvement, and is closer to contemporary society in terms of its publication date. In this study, the scale was translated into Chinese to provide a clearer understanding of its contents for the research subjects. The scale consists of 10 items, is unidimensional (no subscales), and aims to measure the overall level of participants' psychological resilience. For each item, the five options “Completely Disagree,” “Occasionally Agree,” “Sometimes Agree,” “Fairly Agree,” and “Strongly Agree” are scored 0 to 4 points, respectively. The total score for all items is summed to obtain the participant's overall psychological resilience score. In this study, the scale's reliability was 0.938 and its validity was 0.964, indicating that the scale reliably and effectively measured the target variable in this study, supporting the scientificity and credibility of the findings (see Table 2).

#### 3.2.4 Career Decision-Making Difficulty Scale (CDMP)

The Career Decision-Making Difficulty Scale (CDMP) was proposed by Chinese scholars Rui and Lirong (2006), localized and revised from the Career Decision-Making Difficulties Questionnaire (CDDQ) developed by Gati et al. (2010), and adjusted based on the actual employment situation of Chinese youth and the characteristics of the Chinese labor market. The scale consists of 16 items, containing four sub-dimensions: Career Information Exploration, Career Self Exploration, Career Planning Exploration, and Career Goal Exploration, aiming to measure participants' career decision-making ability. For each item, the five options “Completely Disagree,” “Occasionally Agree,” “Sometimes Agree,” “Fairly Agree,” and “Strongly Agree” are scored 1–5 points, respectively. The total score for all items is summed to obtain the participant's overall career decision-making ability score. In this study, the scale's reliability was 0.957 and its validity was 0.980, indicating that the scale reliably and effectively measured the target variable in this study, supporting the scientificity and credibility of the findings (see Table 2).

### 3.3 Data processing

SPSS 27.0 software was used to test the reliability and validity of the scales and to perform descriptive statistics on the data collected from the questionnaires. Simultaneously, this study used AMOS 26.0 software to test and refine the constructed chain mediation model.

## 4 Research results

To mitigate risks potentially arising from common method bias (CMB) during data collection, this study implemented controls from two perspectives: First, reverse scoring and scrambling the question order were used to reduce response pattern bias; Second, the anonymity of questionnaire responses was explicitly emphasized to control social desirability bias (Kock et al., 2021). Harman's single-factor test results showed that the variance explained by the first common factor in this study was 37.406%, according to Podsakoff's study, if the variance explained by the first factor is < 50%, it is generally considered that the CMB problem is within an acceptable range and will not pose a serious threat to the research results, which supports the reliability and validity of the research conclusions (Podsakoff et al., 2003).

This study used structural equation modeling (SEM) for hypothesis testing. When evaluating the overall goodness of fit of the model, we mainly refer to the following fitting indices and their recognized critical criteria: the chi square degree of freedom ratio (χ^2^/df) < 3 is acceptable; Root Mean Square Error of Approximation (RMSEA) < 0.08 (90% CI upper limit < 0.08) is acceptable; Standardized root mean square residual (SRMR) < 0.08 is considered good; A Comparative Fit Index (CFI) > 0.90 is considered acceptable; A Tucker Lewis index (TLI) > 0.90 is acceptable. After ensuring the reliability and validity of all measures and addressing potential common method bias, the following section presents the research results.

### 4.1 Confirmatory factor analysis

#### 4.1.1 Structural validity

The results of the model's structural validity test are shown in Tables 3, 4. The combined indicators demonstrate a good fit between the model and the data. In terms of absolute fit indices, the chi-square value (χ^2^ = 747.323, df = 692) had a corresponding *p*-value of 0.071 which may be due to the moderate sample size and model simplicity. And the chi-square/degrees of freedom ratio (χ^2^/df) was 1.08 (between 1 and 3 and < 2), both indicating acceptable model fit and appropriate model complexity. The root mean square residual (RMR) was 0.039 (< 0.05), and the root mean square error of approximation (RMSEA) was 0.014 (much < 0.05), both at excellent levels, indicating small model residuals and high fitting precision. The goodness-of-fit index (GFI = 0.918) and adjusted goodness-of-fit index (AGFI = 0.917) were above 0.90, considered satisfactory; Hoelter's critical N (CN = 415) was far >200, strongly supporting model fit. In terms of incremental fit indices, the normed fit index (NFI = 0.942), relative fit index (RFI = 0.937), incremental fit index (IFI = 0.995), Tucker-Lewis index (TLI = 0.995), and comparative fit index (CFI = 0.995) all met the standard of 0.90, indicating a significant improvement in fit compared to the baseline model. Parsimonious fit indices (PGFI = 0.814, PNFI = 0.879, PCFI = 0.930) were all significantly higher than the acceptable standard of 0.50, indicating that the model maintained good parsimony while achieving excellent fit. In summary, all key indicators reached ideal or acceptable levels, the overall model fit was excellent, strongly supporting the model's validity.

**Table 3:** Absolute fit indices.

**χ^2^**	**DF**	**p**	**χ^2^/DF**	**RMR**	**RMSEA**	**GFI**	**AGFI**	**CN**
747.323	692	0.071	1.08	0.039	0.014	0.918	0.917	415

**Table 4:** Incremental fit and parsimonious fit indices.

**NFI**	**RFI**	**IFI**	**TLI**	**CFI**	**PGFI**	**PNFI**	**PCFI**
0.942	0.937	0.995	0.995	0.995	0.814	0.879	0.930

#### 4.1.2 Convergent validity

The results showed that all scales in the questionnaire exhibited good convergent validity (Table 5). Specifically, for the Physical Activity Rating Scale, the standardized factor loadings of its 3 items ranged from 0.751 to 0.765 (all >0.7), composite reliability CR = 0.803 (>0.7), average variance extracted AVE = 0.576 (>0.5), and all items were statistically significant (*p* < 0.01). The SMC values (0.564–0.585) indicated good explanatory power for each item. For the General Self-Efficacy Scale, the standardized loadings of its 10 items were also well above 0.7 (0.846–0.896), CR reached 0.968, AVE = 0.749, and SMC values (0.716–0.803) showed strong explanatory power for each item; *t*-values were all >22.4 (*p* < 0.01). For the Psychological Resilience Scale, the standardized loadings of its 10 items ranged from 0.744 to 0.809, CR = 0.939, AVE = 0.604, all items were significant (*t* > 16.4, *p* < 0.01), and SMC values ranged from 0.554 to 0.654. The Career Decision-Making Difficulty Scale was divided into four sub-dimensions: For Career Information Exploration (5 items), loadings ranged from 0.736 to 0.774 (CR = 0.874, AVE = 0.580); for Career Self-Exploration (4 items), loadings ranged from 0.737 to 0.775 (CR = 0.848, AVE = 0.583); for Career Planning Exploration (3 items), loadings ranged from 0.749 to 0.782 (CR = 0.810, AVE = 0.586); for Career Goal Exploration (4 items), loadings ranged from 0.737 to 0.772 (CR = 0.841, AVE = 0.569). All items had *t*-values >15.5 (*p* < 0.01) and SMC values >0.5. In summary, the measurement indicators for all latent variables met the convergent validity criteria (standardized loadings >0.7, CR > 0.7, AVE > 0.5), and all scales demonstrated excellent internal consistency and item representativeness. These results confirm satisfactory convergent validity for all constructs.

**Table 5:** Convergent validity indices.

**Dimension**	**Indicator**	**Std**.	**Unstd**.	**S.E**.	**C.R (*t*-value)**	**SMC**	**CR**	**AVE**
Physical activity level	PARS-3 q1	0.761	1			0.579	0.803	0.576
Physical activity level	PARS-3 q2	0.765	1.028	0.078	13.26	0.585		
Physical activity level	PARS-3 q3	0.751	1.027	0.078	13.162	0.564		
Self-efficacy	GSES q1	0.85	1			0.723	0.968	0.749
Self-efficacy	GSES q2	0.863	1.024	0.044	23.335	0.745		
Self-efficacy	GSES q3	0.871	1.051	0.044	23.702	0.759		
Self-efficacy	GSES q4	0.863	1.037	0.044	23.363	0.745		
Self-efficacy	GSES q5	0.873	1.065	0.045	23.827	0.762		
Self-efficacy	GSES q6	0.854	1.014	0.044	22.904	0.729		
Self-efficacy	GSES q7	0.86	1.027	0.044	23.164	0.740		
Self-efficacy	GSES q8	0.846	0.989	0.044	22.448	0.716		
Self-efficacy	GSES q9	0.896	1.097	0.044	25.047	0.803		
Self-efficacy	GSES q10	0.878	1.069	0.044	24.127	0.771		
Psychological resilience	CD-RISC-10 q1	0.791	1			0.626	0.939	0.604
Psychological resilience	CD-RISC-10 q2	0.788	1.016	0.057	17.774	0.621		
Psychological resilience	CD-RISC-10 q3	0.785	0.951	0.054	17.626	0.616		
Psychological resilience	CD-RISC-10 q4	0.787	0.988	0.056	17.755	0.619		
Psychological resilience	CD-RISC-10 q5	0.751	0.939	0.056	16.673	0.564		
Psychological resilience	CD-RISC-10 q6	0.809	1.026	0.056	18.364	0.654		
Psychological resilience	CD-RISC-10 q7	0.761	0.964	0.057	16.874	0.579		
Psychological resilience	CD-RISC-10 q8	0.796	1.025	0.057	17.965	0.634		
Psychological resilience	CD-RISC-10 q9	0.76	0.91	0.054	16.918	0.578		
Psychological resilience	CD-RISC-10 q10	0.744	0.922	0.056	16.464	0.554		
Career information exploration	CDMP q1	0.753	1			0.567	0.874	0.580
Career information exploration	CDMP q5	0.736	0.978	0.063	15.632	0.542		
Career information exploration	CDMP q8	0.772	1.051	0.063	16.565	0.596		
Career information exploration	CDMP q11	0.773	1.055	0.064	16.579	0.598		
Career information exploration	CDMP q14	0.774	1.019	0.061	16.57	0.599		
Career self exploration	CDMP q2	0.737	1			0.543	0.848	0.583
Career self exploration	CDMP q6	0.775	1.083	0.067	16.202	0.601		
Career self exploration	CDMP q9	0.775	1.088	0.067	16.209	0.601		
Career self exploration	CDMP q12	0.766	1.047	0.066	15.961	0.587		
Career planning exploration	CDMP q3	0.782	1			0.612	0.810	0.586
Career planning exploration	CDMP q7	0.766	0.969	0.057	16.885	0.587		
Career planning exploration	CDMP q15	0.749	0.936	0.057	16.391	0.561		
Career goal exploration	CDMP q4	0.746	1			0.557	0.841	0.569
Career goal exploration	CDMP q10	0.772	1.073	0.066	16.358	0.596		
Career goal exploration	CDMP q13	0.762	1.008	0.063	16.102	0.581		
Career goal exploration	CDMP q16	0.737	0.947	0.061	15.501	0.543		

*p* < 0.01.

#### 4.1.3 Discriminant validity

The discriminant validity analysis results (Table 6) showed that all latent variables satisfied the Fornell-Larcker criterion, indicating good discriminant validity of the scales (Fornell and Larcker, 1981). In the model, the square root of the AVE for Physical Activity was 0.759, significantly greater than its correlation coefficients with Self-Efficacy (*r* = 0.350), Psychological Resilience (*r* = 0.398), and Career Decision-Making Behavior (*r* = 0.399). The square root of the AVE for Self-Efficacy was 0.865, far exceeding its correlations with Psychological Resilience (*r* = 0.327) and Career Decision-Making Behavior (*r* = 0.376). The square root of the AVE for Psychological Resilience was 0.777, higher than its correlation with Career Decision-Making Behavior (*r* = 0.450). The square root of the AVE for Career Decision-Making Behavior was 0.761, also greater than all correlation coefficients between variables. Since the square root of the AVE for each latent variable was greater than the corresponding correlation coefficients in its row and column, and all correlation coefficients were < 0.5, it indicates that each construct is effectively related yet maintains independent distinctiveness, fully validating the discriminant validity between the scales.

**Table 6:** Discriminant validity indices.

**Index**	**Physical activity level**	**Self-efficacy**	**Psychological resilience**	**Career decision-making**
Physical activity level	**0.759**			
Self-efficacy	0.350	**0.865**		
Psychological resilience	0.398	0.327	**0.777**	
Career decision-making	0.399	0.376	0.450	**0.761**

The diagonal elements represent the square root of AVE. Bold values indicate average variance extracted.

The above confirmatory factor analysis results demonstrate that the model has good structural validity, convergent validity, and discriminant validity, indicating that the scales possess excellent measurement quality and the research data support the theoretical framework. Simultaneously, this study's model provides a foundation for subsequent causal inference and analysis.

### 4.2 Descriptive statistics and correlation analysis

Descriptive statistics and correlation analysis of the research variables showed that in the effective sample of *n* = 412, the sample's total physical activity score (9.820 ± 2.887), total self-efficacy score (27.610 ± 10.848), total psychological resilience score (33.600 ± 9.172), and total career decision-making behavior score (52.92 ± 14.157) all exhibited significant positive correlations. Physical activity level was moderately positively correlated with self-efficacy (*r* = 0.309, *p* < 0.01), psychological resilience (*r* = 0.345, *p* < 0.01), and career decision-making behavior (*r* = 0.351, *p* < 0.01). Self-efficacy was significantly correlated with psychological resilience (*r* = 0.311, *p* < 0.01) and career decision-making behavior (*r* = 0.361, *p* < 0.01). The strongest correlation was between psychological resilience and career decision-making behavior (*r* = 0.428, *p* < 0.01). Overall, individuals with higher levels of physical activity tended to have higher self-efficacy and psychological resilience, as well as more effective career decision-making behavior. The association between psychological resilience and career development was most prominent among the surveyed subjects in this study (Table 7). All correlations are positive and moderate-to-strong, supporting the model hypotheses.

**Table 7:** Pearson correlation analysis.

**Index**	**Physical activity level**	**Self-efficacy**	**Psychological resilience**	**Career decision-making**	**M ±SD**
Physical activity level	1.000				9.820 ± 2.887
Self-efficacy	0.309^**^	1.000			27.610 ± 10.848
Psychological resilience	0.345^**^	0.311^**^	1.000		33.600 ± 9.172
Career decision-making	0.351^**^	0.361^**^	0.428^**^	1.000	52.92 ± 14.157

^**^p < 0.01.

### 4.3 Structural equation model

**Figure 2:**
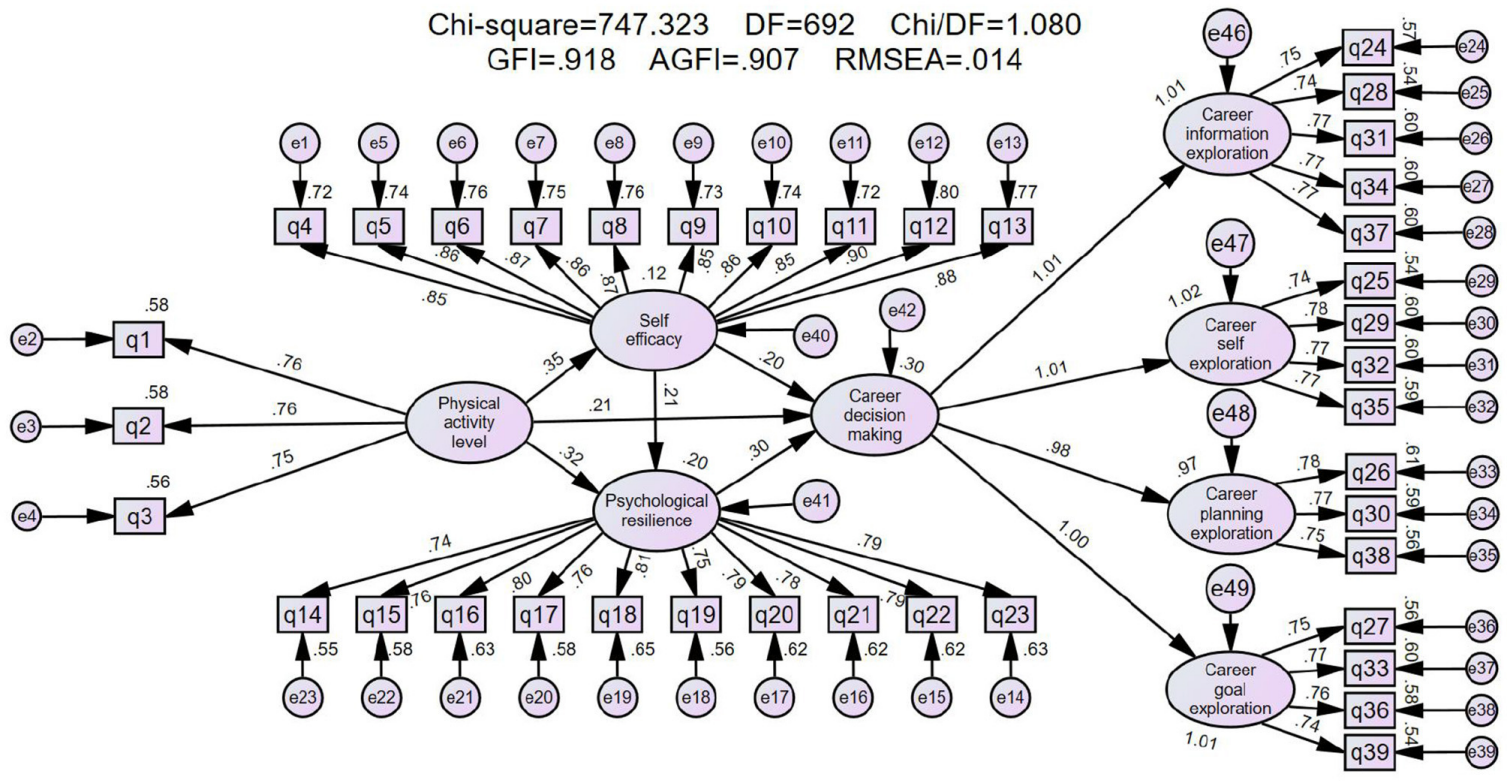
Standardized structural equation model.

Given that the model demonstrated good structural validity, convergent validity, and discriminant validity, and Pearson correlation analysis showed significant associations between all parts of the model, it can be concluded that, within the current sample and measurement context, the structural equation model for the chain mediation effect of self-efficacy and psychological resilience on how physical exercise habits influence individual career decision-making behavior is valid (Figure 2). First, the standardized loading factors in the model were all >0.6. Except for the loading factors between career decision-making behavior and its four sub-dimensions, all other standardized loading factors ranged from 0.6 to 0.95, indicating excellent ability of each item to explain the latent variables. Second, the SMC values of the measurement model ranged from 0.5 to 0.9, indicating high reliability quality of the observed variables and strong explanatory power of the latent variables. Finally, given that the path coefficients between latent variables in the unstandardized path coefficients (Table 8) were all significant (*p* < 0.01), the magnitude of the path coefficients in the structural model was examined. In this study, the path Physical Activity to Self-Efficacy (β = 0.425) and physical activity to Psychological resilience (β = 0.348) was moderate, while other paths were weaker (β < 0.3).

**Table 8:** Non-standardized path coefficients.

**Path**	**Estimate**	**S.E**.	**C.R**.	***P***
Physical activity level → self-efficacy	0.425	0.069	6.138	*p* < 0.01
Physical activity level → psychological resilience	0.348	0.064	5.41	*p* < 0.01
Self-efficacy → psychological resilience	0.19	0.047	4.075	*p* < 0.01
Physical activity level → career decision-making	0.209	0.058	3.627	*p* < 0.01
Self-efficacy → career decision-making	0.169	0.041	4.1	*p* < 0.01
Psychological resilience → career decision-making	0.279	0.05	5.589	*p* < 0.01

### 4.4 Chain mediation effect

To further confirm the existence of the chain mediation effect of self-efficacy and psychological resilience on the influence of physical exercise habits on career decision-making behavior, the study further employed the Bootstrap sampling method (5,000 times) to test the mechanism through which physical activity level affects career decision-making behavior (Table 9). Setting participants' physical activity level score as the independent variable and career decision-making behavior score as the dependent variable, with self-efficacy and psychological resilience scores as mediating variables, the results revealed significant multiple impact pathways. First, physical activity had a direct positive impact on career decision-making behavior, with a standardized effect size of 0.169 [95% CI [0.123, 0.217]]. Second, the independent mediating effect of self-efficacy between the independent and dependent variables was 0.066 [95% CI [0.035, 0.100]]. Third, the independent mediating effect of psychological resilience between the independent and dependent variables was 0.082 [95% CI [0.049, 0.119]]. Fourth, the chain mediation path effect via “Self-Efficacy → Psychological Resilience” between the independent and dependent variables was 0.021 [95% CI [0.011, 0.034]]. The 95% confidence intervals for all paths did not include zero, confirming significant effects. The mediating strength of psychological resilience exceeded that of self-efficacy (0.082 > 0.066), and the direct effect dominated the total effect (~50%). The three mediating paths together constituted nearly 40% of the influence mechanism, revealing that physical exercise habits can influence individuals' career decision-making behavior through the chain mediation effect of self-efficacy and psychological resilience. These findings suggest that promoting physical activity can improve career decision-making mainly by strengthening psychological resources, even though the direct effect remains dominant (~50%). These results provide strong evidence for discussing the role of psychological variables in linking physical activity and career decision-making, as elaborated in the following discussion.

**Table 9:** Chain mediation effects.

**Impact path**	**Std. effect (boot)**	**95% CI**	

		**LL**	**UL**
Physical activity level → career decision-making	0.169	0.123	0.217
Physical activity level → self-efficacy → career decision-making	0.066	0.035	0.100
Physical activity level → psychological resilience → career decision-making	0.082	0.049	0.119
Physical activity level → self-efficacy → psychological resilience → career decision-making	0.021	0.011	0.034

## 5 Discussion

### 5.1 The central role of psychological capital

Through a rigorous chain mediation model, this study systematically reveals the complex psychological mechanism through which physical exercise habits profoundly influence college students' career decision-making behavior. Its core findings can be theoretically explained in depth from three dimensions.

#### 5.1.1 Direct effect of physical exercise on career decision-making

The relevant path coefficient (β = 0.169, *p* < 0.01) strongly confirms the direct enhancing effect of physical exercise habits on career decision-making ability. This finding not only aligns closely with conclusions by scholars like Ouyang regarding exercise enhancing cognitive flexibility and optimizing problem-solving abilities (Ouyang et al., 2020), but also deeply reveals the embodied cognitive connection between physical activity and career competence. Ouyang found that the degree of individual participation in sports is significantly positively correlated with their self-efficacy, and self-efficacy plays a significant mediating role in the relationship between individual body image and sports participation. Analyzing from the perspective of cross-contextual transfer of goal execution, it becomes evident that the specific goals set in sports training and their implementation process essentially constitute a complete cycle of plan “formulation-execution-monitoring adjustment.” This embodied goal management experience directly transfers to the ability to set and execute career goals, making individuals more systematic and persistent when facing career choices.

Secondly, team sports like basketball create micro-social systems that require participants to instantly interpret teammates' intentions, coordinate actions, and adapt to dynamic roles, strengthening individuals' social learning for situational adaptation. This immersive collaborative experience can also significantly enhance an individual's ability to interpret situations and generate adaptive behaviors in complex workplace environments. Furthermore, viewing sports competitions as training grounds for frustration tolerance, the inevitable loss situations in sports provide low-risk, high-frequency scenarios of exposure to setbacks. By repeatedly experiencing the process of reflection and reattempt after failure, individuals' anxiety threshold for potential failures in career decision-making increases, effectively reducing decision avoidance behaviors.

#### 5.1.2 Validation of dual independent mediation paths

The study innovatively validated two coexisting and independent mediation paths, revealing the differentiated psychological processes through which physical exercise influences career decision-making. Regarding the path with self-efficacy as an independent mediator (β = 0.066, *p* < 0.01), this path perfectly illustrates the cross-domain generalization mechanism of Bandura's self-efficacy theory (Bandura and Wessels, 1997). The experiences of conquering physical challenges accumulated through physical exercise are metaphorically mapped through embodied cognition, such as “I can conquer this mountain → I can overcome this career challenge,” transforming into a generalized belief in one's ability to handle career challenges (Hirschi et al., 2015). This enhanced self-efficacy directly improves the efficiency of career information search, evaluation, and integration, making individuals more confident and proactive in decision-making. The path with psychological resilience as an independent mediator (β = 0.082, *p* < 0.01) provides empirical support for the physiological-psychological linkage model proposed by Qiu et al. (2025).

On one hand, it is speculated in this study that the release of neurobiochemical substances like endorphins and BDNF (Brain-Derived Neurotrophic Factor) induced by regular exercise directly reduces anxiety levels physiologically, creating an emotional state more conducive to rational decision-making, which was not measured in this study due to limitations of research methodology. On the other hand, the persistence required by long-term sports training essentially systematically elevates the stress tolerance threshold. This pressure resistance forged at the physical level directly transfers into psychological elasticity when facing career uncertainty, significantly reducing decision-making procrastination caused by fear of difficulties (Parola and Marcionetti, 2022).

#### 5.1.3 Core value of chain mediation

The study revealed the chain path “Physical Exercise → Self-Efficacy → Psychological Resilience → Career Decision-Making” (β = 0.021, *p* < 0.01), not only proving the sequential linkage of multiple psychological traits but also clarifying the internal logic of their synergistic enhancement. Students with higher self-efficacy and resilience are more likely to persist in internships or competitive job applications despite setbacks. Individuals with high self-efficacy do not ignore stress but are adept at using positive cognitive reappraisal strategies (Santos et al., 2018). They perceive career decision-making pressure as an opportunity to prove their abilities or a catalyst for growth, rather than a threat. This active cognitive reframing lays the cognitive foundation for the manifestation of psychological resilience. The core value of psychological resilience becomes particularly prominent when individuals encounter real setbacks. At this point, high self-efficacy alone may waver due to setbacks, while psychological resilience demonstrates stability and buffering during behavioral execution, ensuring that the positive decision-making intentions driven by self-efficacy can be continuously translated into actual actions, avoiding behavioral interruption or giving up halfway (Fletcher, 2013). The chain effect indicates that self-efficacy triggers the individual's willingness to act, while their psychological resilience ensures the endurance of that action in adversity.

##### 5.1.3.1 Core findings

(1) Direct effect: Physical exercise significantly improves career decision-making ability (β = 0.169), achieved through enhanced goal management, collaborative experience, and resilience to setbacks.
(2) The independent mediating effect of self-efficacy (β = 0.066): Exercise experience enhances occupational ability beliefs through embodied cognitive generalization.
(3) The independent mediating effect of psychological resilience (β = 0.082): The transfer of resilience in exercise behavior buffers occupational decision-making pressure.
(4) Chain mediated value: Self-efficacy → Psychological resilience pathway (β = 0.021) reveals the temporal synergy of psychological traits: Self-efficacy triggers action intention, while psychological resilience ensures action persistence in adversity.

### 5.2 Theoretical value and practical implications

#### 5.2.1 Theoretical value

This study supplements and improves existing research at the theoretical level. Traditional career decision-making research mainly focuses on pure cognitive variables such as career interest assessment, information processing models, and cognitive style analysis, relatively neglecting the fundamental role of non-cognitive psychological traits and physiological foundations (Hirschi et al., 2015). This study provides conclusive evidence that physical exercise, as a powerful physiological activation and behavior shaping tool, indirectly and effectively optimizes career decision-making behavior and outcomes by efficiently cultivating the core components of psychological capital, namely self-efficacy and psychological resilience. This discovery challenges the traditional assumptions of the detached mind and provides a solid embodied cognitive perspective for understanding career decision-making, demonstrating that physical experience can systematically reshape the psychological framework of decision-making. Compared to previous studies that generally relied on a single mediation model, this study, by verifying the chain mediation effect, for the first time clearly reveals the temporal linkage between self-efficacy and psychological resilience in the field of career decision-making. The dynamic synergistic mechanism of psychological traits revealed by research indicates that self-efficacy and psychological resilience are not simply additive, but form a pool of psychological resources that can be activated and called upon as a whole. The combined effect significantly exceeds the sum of their independent effects, resulting in a synergistic gain. This strongly confirms the argument of Pang et al. that psychological capital, as a higher-order core concept, has a much greater integrated value than simply adding up its components (Pang et al., 2021).

#### 5.2.2 Practical implications

The results of this study provide an operable practical path for colleges and universities to optimize the career education system. At the institutional level: (1) promote the in-depth integration of career education and physical education curriculum Colleges and universities should develop diversified physical education curriculum modules, integrate professional ability training (such as team cooperation and pressure resistance) into sports experience, and enable students to naturally improve their psychological capital (such as resilience and self-confidence) in physical activities. (2) Establish a dynamic psychological file system Colleges and universities need to build a dynamic system related to students' sports participation data, psychological resource evaluation (such as resilience, optimism scale) and the evaluation results of career decision-making ability. The system should be able to identify low resilience groups and provide data support for the school to develop accurate group or individual intervention programs (such as targeted exercise prescriptions and workshops). (3) Reconstruct the function of campus support system Colleges and universities should guide sports community activities to integrate career development elements. For example, the risk decision-making simulation link is integrated into the design of the outdoor adventure Association, or the project management ability such as budget formulation, task division, schedule control is clearly designed and cultivated in the event planning community activities.

The key points of optimizing policy implementation are: (1) reform the evaluation system of physical education curriculum. The incremental changes of students' psychological capital (such as self-confidence and sense of purpose) are included in the evaluation index to replace or weaken the single skill assessment. (2) Improve resource accessibility. For example, by significantly extending the opening hours of sports facilities such as gymnasiums in the morning (e.g., to 6:30 in advance) and at night (e.g., to 22:00), we can adapt to the fragmented schedule of students and reduce the threshold of participation.

In terms of students' individual application, students can take the initiative to use the above integrated resources provided by the school to participate in structured sports activities as a direct way to improve their professional ability. For example, students can consciously join structured group sports such as basketball and volleyball teams. In regular training and competition, by constantly coping with instant decision-making, dealing with the pressure of victory and defeat, and cooperating with teammates to solve conflicts, their decision-making self-confidence and on-site adaptability will be directly trained and significantly improved. This successful experience in a safe and supportive environment can be directly transferred to the career decision-making scene to enhance their confidence in making and adhering to career choices.

##### 5.2.2.1 Theoretical breakthrough

(1) Challenge the “cognitive segregation hypothesis” in career decision-making research and establish an embodied cognitive path of physical exercise → psychological capital → career decision-making.
(2) The first verification of the chain synergy mechanism between self-efficacy and psychological resilience (not a simple superposition) supports the higher-order integration value of psychological capital.

##### 5.2.2.2 Practical path

(1) Course integration: Incorporating team collaboration/stress resistance training into physical education classes naturally enhances psychological capital.
(2) Dynamic psychological profile: linking sports participation, psychological resources, and decision-making ability data to accurately identify low resilience groups.
(3) Support system refactoring: Sports clubs integrate career development elements (such as simulating risk decision-making, project management training).

### 5.3 Limitations and future directions

#### 5.3.1 Research limitations

This study has several limitations that need to be addressed: Firstly, the main limitation of this study lies in its cross-sectional design, which limits the ability to make causal inferences. In addition, the study only measured decision-making behavior without tracking actual employment quality, making it difficult to verify the conversion efficiency of individual decision-making behavior into employment outcomes. Secondly, the sample structure affects the generality of the conclusions. Due to the objective reality of student enrollment proportions in China, the proportion of students majoring in Education (16.67%) and Science & Engineering (23.88%) in the valid sample was significantly higher than those in Arts (7.96%) and Medicine (7.71%), potentially weakening the explanatory power of this study's model for some student groups. Due to the fact that the PARS-3 scale and CDMP scale are Chinese modified versions, the research results may lack international universality. Thirdly, the depth of mechanism analysis is insufficient. The differential effects of exercise types were not distinguished; for instance, team sports might enhance collaborative self-efficacy, while endurance training might more directly enhance stress resilience. There was also a lack of empirical testing of physiological and biochemical indicators such as salivary cortisol or serum BDNF concentration. In addition, it is speculated that potential COVID-19 or other environmental factors may also affect individual physical exercise habits. Finally, this study relies on a self-administered questionnaire to collect core variables, which may result in self-report bias.

#### 5.3.2 Future directions

Based on the above limitations, future research can deepen in three aspects: First, refined exercise intervention experiments should be conducted. Randomized Controlled Trial (RCT) designs could be used to group students with low career decision-making ability scores into different exercise program groups and a control group, tracking changes in career decision-making indicators after 8 weeks to analyze the specific pathways of different exercise types. Following students from university to the workplace is a follow-up research program that can be considered. At the same time, the interview method is also worth applying during this period. Secondly, the impact of exercise on psychological capital and career decision-making under different cultural backgrounds could be compared to identify the moderating effect of cultural factors. Finally, it is recommended to develop technology-enabled dynamic monitoring tools integrating physiological data from fitness trackers, decision-making behavior logs, and workplace simulation systems to establish a multi-dimensional real-time response model from physiological factors through psychological factors to behavioral factors, providing data support for building personalized career guidance processes. Given the limitations of self-reported data, the conclusion should be considered as exploratory association evidence rather than causal confirmation. Future research suggests combining objective measurements such as biomarkers and device monitoring data for validation.

##### 5.3.2.1 Current limitations

(1) The cross-sectional design limits causal inference and does not track actual employment quality.
(2) The universality of the sample needs to be verified.
(3) Lack of differentiation analysis of exercise types (such as team vs. endurance exercise) and empirical evidence of physiological indicators (cortisol, BDNF).

##### 5.3.2.2 Future direction

(1) Refined exercise intervention experiment, grouping tests the improvement path of different types of exercise on career decision-making ability.
(2) Cross cultural comparison to examine the moderating effect of cultural factors on the chain of “sports psychological capital career decision-making.”
(3) Technology empowers monitoring, integrating wearable device physiological data, decision behavior logs, and workplace simulation systems.

### 5.4 Integrated contributions

This study confirms that physical exercise enhances career decision-making through a sequential chain of self-efficacy and psychological resilience (39.6% mediation). Theoretically, it establishes an embodied cognition path challenging cognitive-centric frameworks. Practically, it proposes actionable strategies: embedding career skill training in physical courses, establishing dynamic psychological profiles, and redesigning sports communities with decision-making simulations collectively forming a bio-psycho-behavioral intervention framework for universities.

## 6 Conclusion

Using structural equation modeling and the Bootstrap method, this study systematically revealed the mechanism through which physical exercise habits influence college students' career decision-making behavior. First, physical exercise habits have a significant positive promoting effect on college students' career decision-making behavior [β = 0.169, 95% CI [0.123, 0.217]], confirming that physical activity can directly strengthen career decision-making behavior by enhancing factors such as goal management ability, collaborative experience, and frustration tolerance. This finding is consistent with previous studies on motor and cognitive and behavioral outcomes. Second, independent mediation paths were established. Self-efficacy plays an independent mediating role between physical exercise and career decision-making behavior [β = 0.066, 95% CI [0.035, 0.100]], indicating that exercise experience can enhance individuals' belief in their career abilities through embodied cognitive generalization. Therefore, sports can be used to enhance the professional confidence of students with low self-efficacy. Simultaneously, the independent mediating effect of psychological resilience was significant [β = 0.082, 95% CI [0.049, 0.119]], revealing that physical exercise can buffer career decision-making pressure through the transfer of behavioral resilience. Students with higher resilience due to regular physical activity are more likely to make timely career decisions under stress. Third, a chain mediation mechanism exists. Self-efficacy and psychological resilience form the chain transmission path “Physical Exercise Habits → Self-Efficacy → Psychological Resilience → Career Decision-Making Behavior” [β = 0.021, 95% CI [0.011, 0.034]], indicating sequential synergy between self-efficacy and psychological resilience. Together, they constitute a mediation mechanism accounting for 39.6% of the total effect. As a result, when carrying out psychological intervention on students, priority should be given to improving their sense of self-efficacy, and then cultivating their psychological resilience. In conclusion, both direct and indirect influences provide a comprehensive understanding of the path, and comprehensive intervention is recommended in the practice of psychological intervention. These results support integrating physical activity into career guidance programs to strengthen students' decision-making abilities.

## Data Availability

The raw data supporting the conclusions of this article will be made available by the authors, without undue reservation.
